# Hypoxia-Targeted Drug Q6 Induces G2-M Arrest and Apoptosis via Poisoning Topoisomerase II under Hypoxia

**DOI:** 10.1371/journal.pone.0144506

**Published:** 2015-12-09

**Authors:** Linlin Chang, Xiaowen Liu, Dandan Wang, Jian Ma, Tianyi Zhou, Ying Chen, Rong Sheng, Yongzhou Hu, Ying Du, Qiaojun He, Bo Yang, Hong Zhu

**Affiliations:** 1 Zhejiang Province Key Laboratory of Anti-Cancer Drug Research, College of Pharmaceutical Sciences, Zhejiang University, Hangzhou, China; 2 ZJU-ENS Joint Laboratory of Medicinal Chemistry, College of Pharmaceutical Sciences, Zhejiang University, Hangzhou, China; 3 Department of Gastroenterology, Sir Run Run Shaw Hospital, School of Medicine, Zhejiang University, Hangzhou, China; German Cancer Research Center, GERMANY

## Abstract

In spite of the tremendous efforts dedicated to developing hypoxia-activated prodrugs, no agents yet have been approved for clinical therapy. In the present study, the hypoxic selective anti-cancer activity as well as the cellular target of a novel tirapazamine (TPZ) analogue, 7-methyl-3-(3-chlorophenyl)-quinoxaline-2-carbonitrile 1,4-dioxide (Q6) were investigated. Q6 implemented anti-cancer effects via poisoning topoisomerase II (topo II) under hypoxia. Modified trapped in agarose DNA immunostaining (TARDIS) assay showed more topo II–DNA cleavage complexes trapped by Q6 than TPZ at even lower concentration. In addition, by introducing ataxia-telangiectasia-mutated (ATM) kinase inhibitors caffeine and KU-60019, we displayed that Q6-triggered apoptosis was attributed, at least partially, to DNA double-strand breaks generated by the topo II-targeting effect. Collectively, Q6 stood out for its better hypoxia-selectivity and topo II-poisoning than the parental compound TPZ. All these data shed light on the research of Q6 as a promising hypoxia-activated prodrug candidate for human hepatocellular carcinoma therapy.

## Introduction

A hallmark of solid tumor is hypoxia, which partially attributes to the outgrowth of cancer cells. Mounting evidences indicate that hypoxia confers highly resistance to conventional chemotherapy and radiation therapy. In addition, hypoxia is thought to promote invasiveness and metastasis, usually correlated with poor patient prognosis. As a physiological feature of solid tumor, hypoxia has also shed light on targeting therapy, namely, developing hypoxia-activated prodrugs (HAPs). HAPs predominantly share a common mechanism that can be reduced to covalent modifiers of DNA in hypoxic cells [1], exhibiting toxic side effects to hypoxic cells and reduced side effects to normoxic cells.

To date, a lot of HAPs have been developed, which can be divided into four classes, including nitro(hetero)-cyclic compounds, N-oxides, quinones, and metal complexes. Notably, tirapazamine (TPZ), which belongs to N-oxides, is one of the first promising HAPs. Although TPZ exhibited promising anti-cancer activity in animal models, the therapeutic effects obtained from phase III clinical trials are limited[2]. Since there is no registered agents being used in clinical therapy, the development of novel hypoxic-selective drug candidates with superior anti-cancer activities still has a long way to go.

Previously, our groups have synthesized a serious of 3-arylquinoxaline-2-carbonitrile 1, 4-Di-N-oxide analogs of TPZ, some of which showed superior antiproliferative activity and hypoxia selectivity to various tumor cell lines[3]. Of these compounds, Q6 has drawn much attention with regard to antitumor activity and particularly hypoxia selectivity, both in vivo and in vitro[3,4]. As a promising candidate for hypoxic selective anti-tumor agent, we have demonstrated that Q6 reduced HIF-1α protein via autophagy–lysosome pathway, which partially contributed to its biological activity[4]. It is noteworthy that, HIF-1α plays crucial roles in angiogenesis, proliferation, antiapoptosis[5,6]. Those agents that only disrupt cellular expression or function of HIF-1α may not possess the ability to kill cancer cells directly. Thus, we could not exclude the possibility that in addition to the HIF-1α suppression, some other mechanism(s) or target(s) may contribute to the anti-cancer activities exerted by Q6.

Most anticancer drugs can induce DNA damage leading to DNA double-strand breaks (DSBs) formation, which can account for the cytotoxicity and cell cycle interference of the drugs directly. DNA DSBs can arise from abortive topoisomerase activity, which undertakes responsibility for resolving the unique problems of DNA entanglement in transcription, replication, chromosome condensation and decondensation[7]. Given the evidences revealed by Peters KB and Brown JM[8], in hypoxia, TPZ, the parental compound of Q6, belongs to topo II poisons which includes several important clinically used drugs such as etoposide and adriamycin (doxorubicin). On the basis of selective anti-cancer effects of Q6 in hypoxia, we investigated its targeting effects on topo II, and the subsequent biological consequences including DNA DSBs, cell cycle, and apoptosis.

## Materials and Methods

### Compounds

Q6 was supplied by Professor Yong-zhou Hu (Zhejiang University, Hangzhou, China)[3]. TPZ (tirapazamine) was purchased from Topharman Shanghai Co. Ltd.. Etoposide (VP16), KU-60019 and caffeine were all purchased from Sigma (St. Louis, MO). Q6, TPZ, VP16 KU-60019 were dissolved in DMSO as stock solutions. Caffeine was dissolved in sterilized water. The stock solutions were kept frozen in aliquot at −20°C and thawed immediately before each experiment.

### Cell culture and establishment of hypoxia culture condition

Three human hepatocellular carcinoma (HCC) cell lines were employed. SMMC-7721, Bel-7402 cells were maintained in RPMI-1640 (Gibco, Grand Island, NY, USA). HepG2 cells were maintained in DMEM (Gibco, Grand Island, NY, USA). All media were supplemented with 10% heat-inactivated fetal bovine serum (FBS, Gibco, Grand Island, NY, USA) plus 2 mM glutamine and 50 unit/ml penicillin. All cell lines were purchased from the Shanghai Institute of Biochemistry and Cell Biology, Chinese Academy of Medical Sciences (Shanghai, China) and incubated at 37°C in a 5% CO_2_ atmosphere. Hypoxic conditions (1% O_2_) were established in a hypoxia incubator (Forma Scientific, Inc., Marietta, OH) where N_2_ was used to compensate for the reduced O_2_ level.

### Western blot analysis

Protein samples were separated by SDS-PAGE and transferred to PVDF membranes (Millipore, Bedford, UK). Blots were blocked for 1 h in 5% milk/0.1% Tween 20 in phosphate buffered saline (PBS-T) and then incubated with primary antibodies (1: 1000) at 4°C overnight. Blots were then washed three times for 15 min in PBS-T, followed by incubation with secondary antibody (according to different primary antibodies, HRP-conjugated goat anti-mouse, anti-rabbit, and rabbit anti-goat IgG were used (1: 5000, Santa Cruz, Dallas, TX)) in 5% milk/PBS-T for 1 h, and then washed three times for 15 min in PBS-T. The membranes were briefly incubated with ECL detection reagent (Amersham Biosciences, Castle Hill, Australia) to visualize the proteins and were then exposed on X-ray film. Primary antibodies used were as follows: ATM (Cat# 600-401-398), and p-ATM (Cat# 600-401-400) were purchased from Rockland Immunochemical (Gilbertsville, PA, USA); cleavage-caspase-3 (Cat# 9661), cleavage-PARP (Cat# 5625P), γ-H2Ax (Cat# 9718), Bax (Cat# 2772s), and Bcl-2 (Cat# 2870), ATR (Cat# 2790), p-ATR (Cat# 2853S) were from Cell Signaling Technology (Beverly, MA); PARP (Cat# 7150), pro-caspase-3 (Cat# 7272), β-Actin (Cat# 1615), Chk1 (Cat# 377231), p-Chk1 (Cat# 2341), Chk2 (Cat# 8813), p-Chk2 (Cat# 2661) were from Santa Cruz (Dallas, Texas, US).

### Trypan Blue Staining

Cells were plated into six-well plates and exposed to Q6 (1 μM) for 72 hours. Cells were then harvested and resuspended with culture medium at a density of 1×10^5^ cells/mL. 10 μL of cells collected from each group was incubated for 2 min with 10 μL of trypan blue solution (Cat# T8154, Sigma, St. Louis, MO). Unstained live cells and total cells were both counted on a hemocytometer.

### Immunofluorescene (IF) staining

HepG2 cells were exposed to Q6 (50 μM) or TPZ (100 μM) for 1 h. For γ-H2AX foci assessment, the cells were fixed in 4% paraformaldehyde in PBS for 20 min at room temperature followed by three rinses in PBS and permeabilization in 0.1% Triton-X100 (in PBS) for 10 min at 4°C. Next, cells were blocked by 4% BSA and incubated with primary rabbit monoclonal antibody against γH2AX (1:200) for 10 h at 4°C. Following two rinses with PBS, cells were incubated for 1 hour with FITC-conjugated secondary antibody (1:200, A-21206, Invitrogen), and subjected to with DAPI staining (1 μg/mL) and imaged with Leica DMI 400B fluorescence microscope.

### Propidium iodide staining for flow cytometry

Cells were seeded into six-well plates and exposed to compounds for indicated times. Cells were then harvested and washed with PBS, fixed with precooled 70% ethanol at 4°C. Staining went along in PBS containing 40 μg/mL RNase A and 10 μg/mL propidiumiodide (Sigma, St. Louis, MO) in the dark for 30 min. For each sample, at least 1×10^4^ cells were analyzed using an FACSCalibur cytometer (Becton Dickinson, Lincoln Park, NJ).

### DNA-topoisomerase II activity assay

Topo II activity was measured by the ATP-dependent decatenation of kinetoplast DNA (kDNA)[9] or the ATP-dependent relaxed pHOT-1[10]. Firstly, untreated and treated cells were harvested on ice. Next, we separated cytoplasmic fractions from nuclear fractions as the references described[11,12]. Then, we assessed topo II activity in nuclear extracts from cells treated with or without indicated compounds. The topo II activity assay reaction buffer consisting of 50 mmol/L Tris-HCl (pH 7.7), 120 mmol/L KCl, 10 mmol/L MgCl_2_, 1mmol/L ATP, 0.5 mmol/L DTT, 0.5 mmol/L EDTA, and 30 μg/mL bovine serum albumin was mixed with 0.1 μg kDNA (TopoGEN, Inc., Columbus, OH) or pHOT-1 (TopoGEN, Inc., Columbus, OH) in a total volume of 20 μL. After incubation at 37°C for 15 min, the reaction was terminated by the addition of 10% SDS (1 μL). The DNA samples were subjected to electrophoresis in a 1% agarose gel in 1×TAE at 4 V/cm for 2 h.

### Trapped in agarose DNA immunostaining assay

Trapped in agarose DNA immunostaining (TARDIS) assay was done as previously reported with few minor modifications[8,13]. Briefly, untreated or treated cells were harvested and mixed with low-melting gel spreading on slides, followed by placing the slides in lysis buffer containing protease inhibitors. Proteins that were not covalently bound to the DNA were then removed by 1mol/L NaCl containing protease inhibitors. Topo II that covalently bound to DNA of each cells was detected using topo IIα–specific polyclonal antibody (Santa Cruz Biotechnology, Santa Cruz, CA) and Alexa Fluor 488 goat anti-rabbit immunoglobulin G (Molecular Probes, Eugene, OR). DNA was stained with 1 μg/μL DAPI. Images were captured using fluorescence microscope (Leica DMI 4000B).

### Gene transfection and RNAi

Cells were seeded on 6-well plates and transfected 24 h later using Lipofectamine 2000 (Invitrogen, 11668–019), according to the manufacturer’s instructions. Human ATM siRNA and control siRNA were obtained from GenePharma Co. Ltd (Shanghai, China).

### Statistical analysis

Data were presented as mean ± SD, and significance was assessed with the Student’s t-test. Differences were considered significant at *p*< 0.05.

## Results

### Q6 exerts potent antitumor activity and hypoxia-selectivity in 3 HCC cell lines

**Fig 1:**
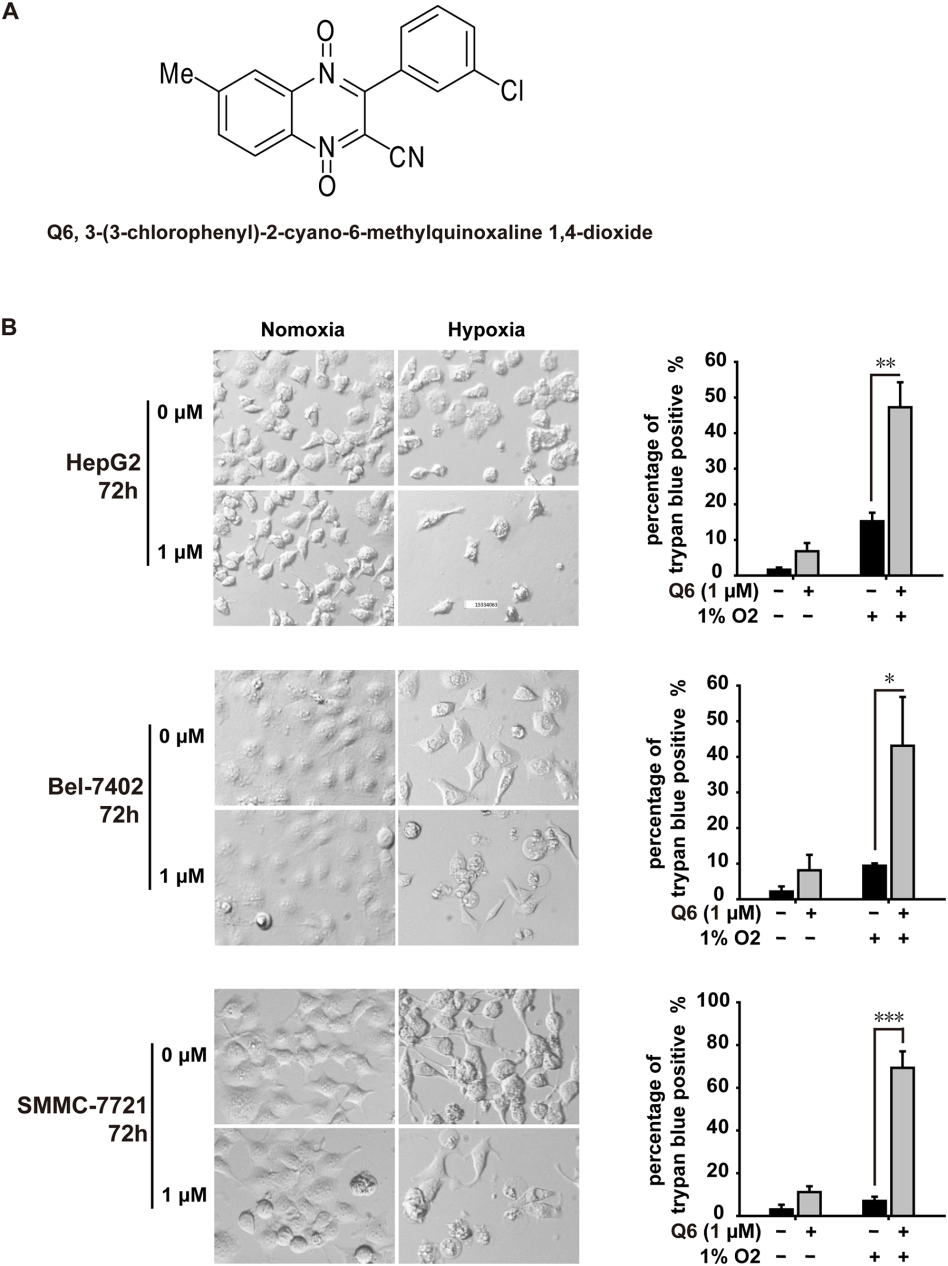
Q6 possessed better anticancer activities under hypoxia. A. The chemical structure of Q6. B. Trypan blue exclusion staining was used to evaluate apoptosis. HepG2, Bel-7402 and SMMC-7721 cells were exposed to Q6 (0, 1 μM) for 72 hours under normoxia and hypoxia, respectively. Percentage of trypan blue positive (%) and representative pictures in different groups were presented.

We have determined the antiproliferative activity of Q6, a TPZ derivative (Fig 1A), against several human cancer cell lines previously[3], suggesting that Q6 may possess considerable anti-tumor characteristics and superior hypoxia-selectivity to TPZ (S2 Fig). Notably, it was evaluated in human hepatocellular carcinoma, with IC_50_ of 2.23 μM for Bel-7402 in hypoxia, 14.7 μM in normoxia, and with IC_50_ of 1.76 μM for HepG2 in hypoxia, 13.1 μM in normoxia. Given the critical contribution of hypoxic microenviroment to HCC maliganancy, Q6 was more potent against HCC cells[4]. In order to further validate the potent activity of Q6 against hypoxic HCC cells, trypan blue exclusion staining assay[14,15] was utilized. As shown in Fig 1B, the exposure to Q6 (1 μM, 72 h) caused much more dead cell poplulation on hypoxia-cultured HCC cell lines HepG2 (47%), Bel-7402(43%) and SMMC-7721 (69%), than that of normoxic HepG2 (7%), Bel-7402(8%) and SMMC-7721 (7%). In addition, those hypoxic HCC cells treated with Q6 exhibit a shrunken and round morphology as compared with those of control groups (0 μM) (Fig 1B). These results suggest that Q6 might be a promising drug candidate for further development as a hypoxic selective anti-tumor agent to treat hepatocellular carcinoma.

### Q6 binds to Topo II spatially

**Fig 2:**
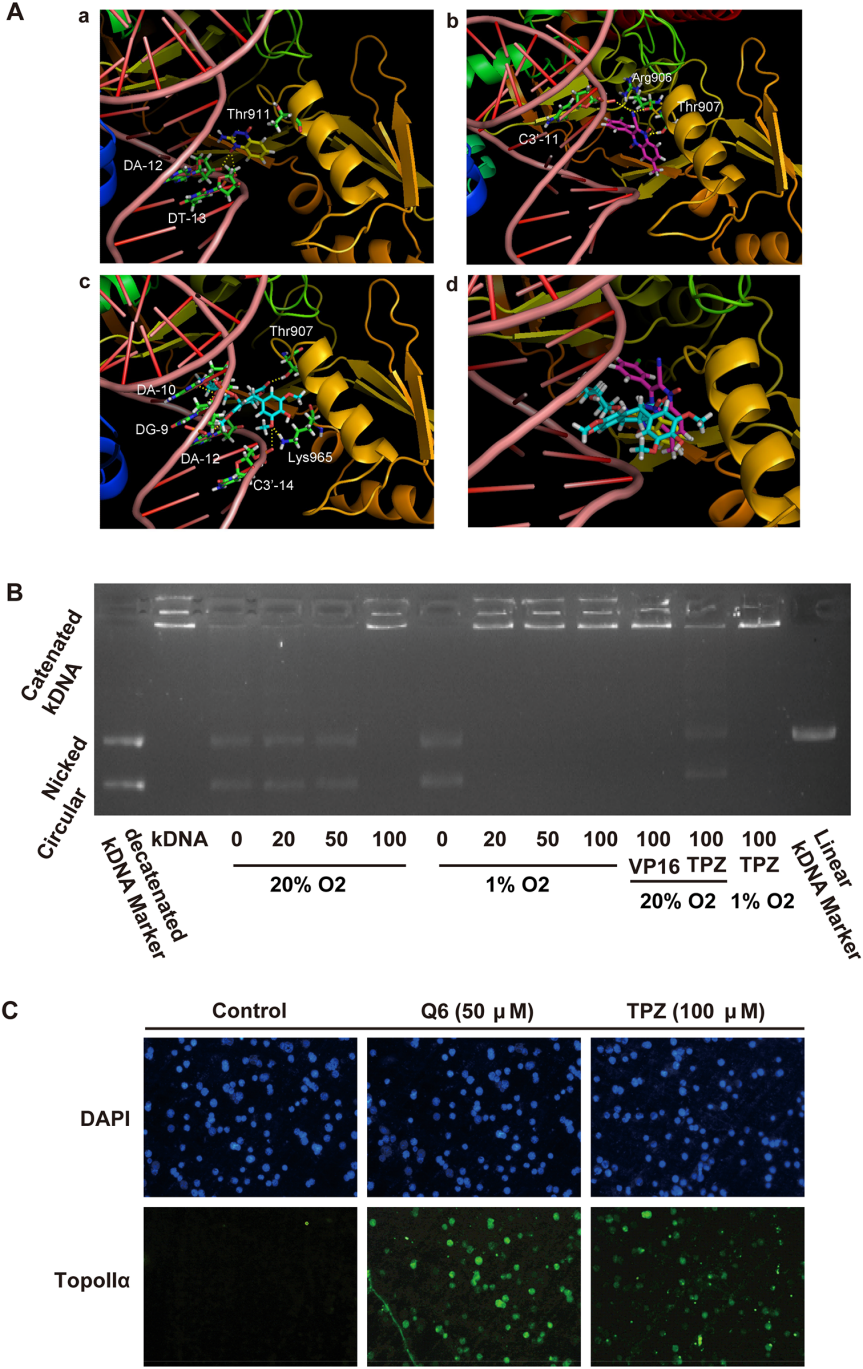
Q6 belonged to topo II poisons. A. Molecular docking of TPZ, Q6, and VP16 with Topoisomerase II (Topo II) -DNA complex. (a) Ribbon show of TPZ docked to Topo II—DNA complex, TPZ is shown in yellow backbone. (b) Ribbon show of Q6 docked to Topo II—DNA complex, Q6 is shown in pink backbone. (c) Surface show of VP16 docked into Topo II—DNA complex, VP16 is shown in light blue backbone. (d) Docking mode comparison among TPZ, Q6 and VP16. Selected residues and nucleobases are shown in green backbone, yellow dashed lines indicate hydrogen bonds, prepared using PyMOL, PDB ID: 2RGR, 3.0 Å. B. Q6 or TPZ, VP16 inhibited topo II—mediated kDNA decatenation from the drug-treated cells as indicated in the figure. C. Detection of DNA-topo II complexes in HepG2 cells using the TARDIS assay. HepG2 cells were treated with either 100 μM TPZ or 50 μM Q6 under hypoxic (1% O_2_) condition for 1 hour. After treatment, cells were suspended in agarose gels on glass slides, lysed, and probed with anti-topo II antibodies.

Since TPZ has been demonstrated to exert anti-cancer activity as a Topoisomerase II (topo II)-targeting agent under hypoxia, the potential effects of Q6 on topo II were explored. Firstly, we used molecular docking technology to examine the binding mode of Q6 with topo II, which was based on the package of Discovery Studio 2.1/CDOCKER, and regarded topo II active site topo II / G section of DNA complex (PDB ID: 2RGR) as a template. The molecular docking analysis (Fig 2A) showed that, quinoxaline basic parent structure of Q6 could effectively bind to topo II and DNA binding region, and form electrostatic interactions with the DNA phosphate groups. Additionally, the quinoxaline ring and 3-chlorophenyl group of Q6 can form effective hydrophobic interactions and hydrogen bondings with topo II amino acid residues (such as Arg 906, Thr 907, etc.), which further reinforced the interactions between Q6 and topo II DNA complexes. As positive controls, TPZ and VP16, the effective inhibitors of topo II, also exhibited effective binding to DNA-topo II complexes in our system.

### Q6 preferentially inhibits the Topo II activity in hypoxic HepG2 cells

Previous studies revealed the hypoxic selectivity of Q6 [3,4] and its interaction with topo II (Fig 2A), we were thus inspired to further investigate that whether Q6 could inhibit the topo II activity, particularly, in those hypoxic cells. HepG2 cells under hypoxia and normoxia were treated with Q6, TPZ or VP16, respectively, followed by nuclear extraction. Given the fact that topo II is predominately located in the nucleus [16], we incubated those nuclear fractions achieved from hypoxia or normoxia agent-exposed cells, with kDNA, the specific substrate for topo II enzyme activity[8]. The kDNA decatenation assay was commonly used to examine the topo II activity [13,17]. Topo II catalyzed the double-stranded catenated kDNA decatenation in the presence of ATP, which generated the minicircles. Inhibition of topo II activity by Q6 was measured as a loss in the capacity to decatenate kDNA. As shown in Fig 2B, in the absence of compounds, kDNA was decatenated to minicircles, which disappeared in a dose-dependent manner in the presence of Q6, and 20 μM Q6 was sufficient to inhibit the activity of topo II in hypoxia as well as 100μM TPZ did. In normoxia both 100 μM Q6 and 100 μM VP16 could inhibit the activity of topo II, whereas 100 μM TPZ only partly weakened the activity of topo II. Similar observation was also achieved from Q6-treated Bel-7402 cells. As shown in S1 Fig, the relaxation of supercoiled pHOT-1 DNA was prohibited by the nuclear extraction from Q6-treated hypoxic Bel-7402 cells. These finding demonstrated that Q6 preferentially target the topo II in those hypoxic cells, and the inhibition activity under hypoxia was stronger than that of TPZ.

### Q6 stabilizes DNA—Topo II complexes

In tradition, topo II—targeted inhibitors are classified as topo II catalytic inhibitors and poisons, and the latter ones were clinical used[18–20]. The superior anti-cancer capacity of poisons were attributed to the formation of topo II-DNA complexes which resulted in the DNA DSBs and the ultimate cell death[18]. In order to specify whether Q6 belongs to topo II poisons or not, the TARDIS assay was carried out to detect the formation of topo II—DNA cleavage complexes at the cellular level (Fig 2C). HepG2 cells were embedded in low melting gel, lysised and washed, incubated with topo IIα primary antibodies and secondary antibodies Alexa Fluor 488 with green fluorescent marked topo II complexed with DNA, then incubated with DAPI (1 μg/μL) for 5 min in the dark. Those dots emitting a green fluorescence and DNA co-localized is topo II cleavable complex. As shown in Fig 2C, 50 μM Q6 under hypoxia emitted much more—green fluorescent compared with the control group as well as TPZ (100 μM) group, suggesting that Q6 possessed the ability to capture and stabilize topo II and DNA formation cleavable complex. These results indicated that Q6 is a novel topo II poison, which may effectively leads to cancer cell death.

### Q6 induces DNA DSBs

**Fig 3:**
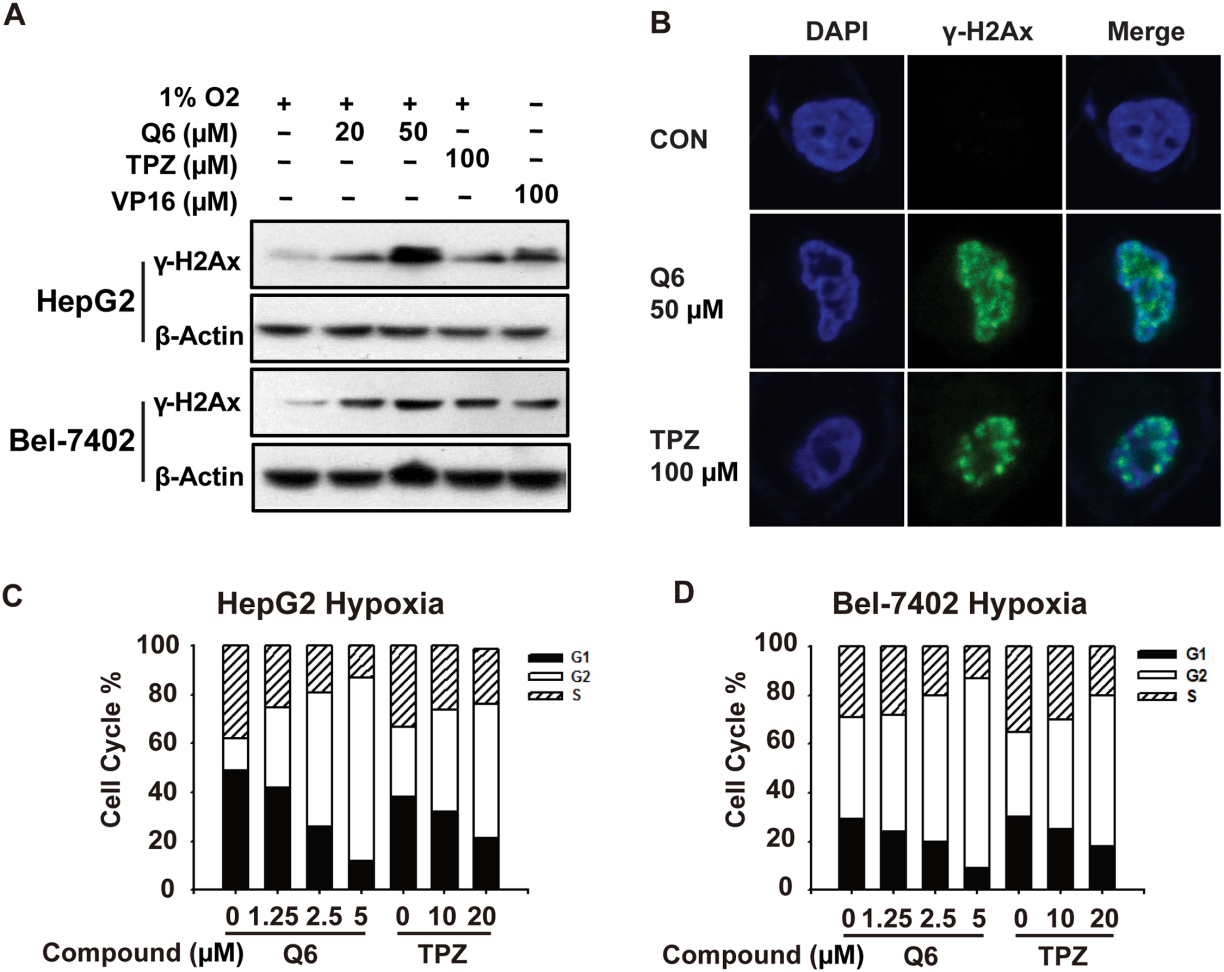
Q6 could induce DNA DSBs followed by G2/M arrest in hypoxia. A. Western blot was employed to assess the DNA DSBs induced by Q6, TPZ, VP16. Protein levels of γ-H2Ax were detected, and β-Actin was measured as the loading control. Data are representative of three independent experiments. B. Immunofluorescene staining was employed to detect the formation of γ-H2AX foci in HepG2 cells. C. and D. Q6 induced G2/M arrest in hepatocellular carcinoma cells. Cells treated with Q6 (1.25 μM, 2.5 μM and 5 μM) or TPZ (10 μM, 20 μM) for 24 h under hypoxia (1% O_2_) condition. Then the cells were collected and prepared for cytometric analysis of cell cycle distribution. Representative cell cycle distribution figures were labeled. The percentages of the cell population in different phases of cell cycle were analyzed by CELL Quest.

Aformentioned data indicated that Q6 could capture and stabilize intermediate DNA-topo II complex, as a novel member of topo II poisons. This process is usually unrecoverable, which leads to DNA double-strand breaks (DSBs) and enables rapid activation of downstream γ-H2AX. Therefore intracellular phosphorylation of H2AX (γ-H2AX) levels can be used as an important indicator of the level of DNA double-strand breaks[21]. For the detection of the extent of DNA DSBs, we firstly employed western blot analysis under hypoxia and normoxia (Fig 3A). The results demonstrated that Q6 (20 μM, 50 μM) significantly enhanced γ-H2AX expression after 1 h-exposure in a dose-dependent manner. And TPZ (100 μM), VP16 (100 μM) can also cause increased γ-H2AX expression. We further detect the foci formation of γ-H2AX to confirm the DNA DSBs generation. As shown in Fig 3B, the γ-H2AX foci formation was monitored and the results showed that Q6 triggered evident γ-H2AX foci formation within 1 h. These results collectively indicated that Q6 can capture topo II cleavable complex and cause DNA DSBs.

### Q6 induces G2-M arrest

Aforementioned data revealed that Q6 caused DNA DSBs under hypoxia, which may due to the topo II targeting effects. Here, flow cytometry was employed to detect the cell cycle distribution of HepG2, Bel-7402 after TPZ and Q6 exposure. Notably, 5 μM Q6 induced 75% G2-M arrest, and 10 μM TPZ induced only 55% G2-M arrest in HepG2 cells for 24 hours (Fig 3B). Similarly, 5 μM Q6 induced 78% G2-M arrest, and 10 μM TPZ induced 62% G2-M arrest in Bel-7402 cells for 24 hours (Fig 3C). Thus, Q6 induces more DSBs-Mediated G2-M arrest than TPZ, which is consistent with its potent activity to trap topo II-DNA complexes and induce DNA DSBs.

### Q6 triggers apoptosis via ATM

**Fig 4:**
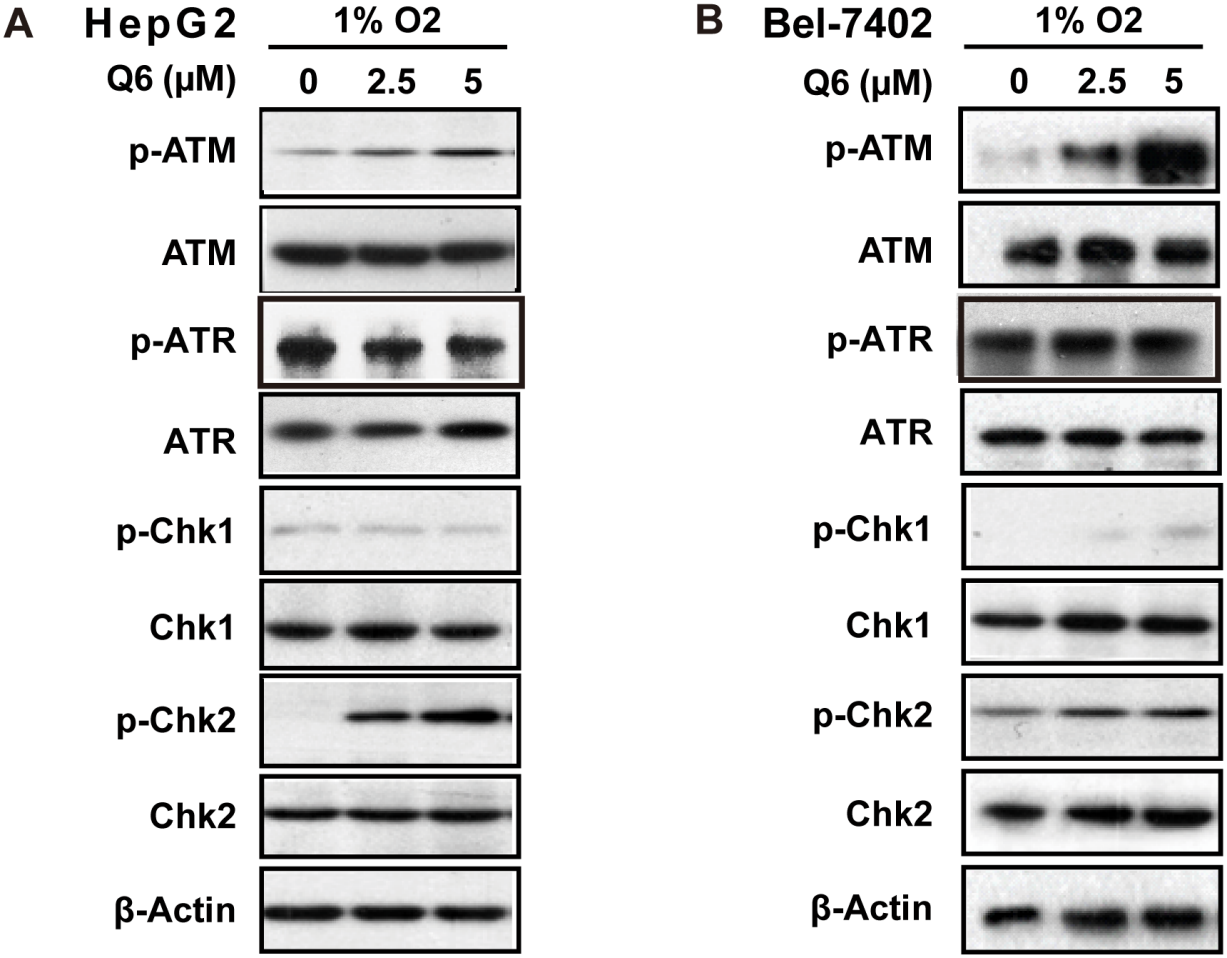
DNA DSBs induced by Q6 triggered ATM-Chk2 pathway in hypoxia. Western blot was carried out to explore the ATM/ATR signaling pathways in respond to DNA DSBs induced by Q6. A. HepG2 cells and B. Bel-7402 cells were treated with different concentration of Q6 (2.5 μM, 5 μM) under hypoxia (1% O_2_) condition. Protein levels were detected by Western blot analysis. β-Actin was measured as the loading control. Data are representative of three independent experiments.

**Fig 5:**
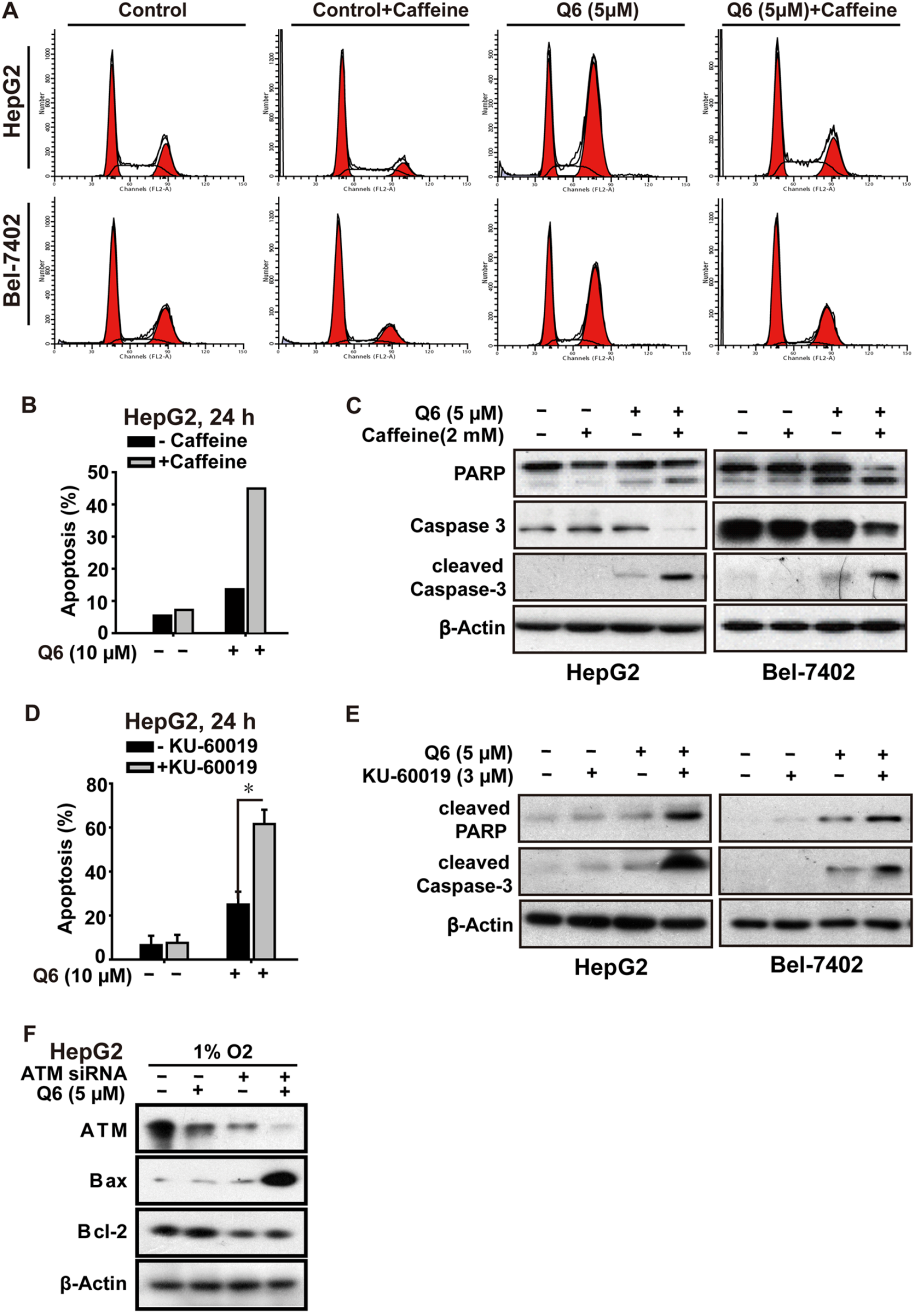
Q6 induced G2/M arrest and apoptosis is ATM/Chk2 dependent in hypoxia. A. HepG2 and Bel-7402 cells, treated with Q6 (5 μM) in the presence or absence of caffeine (2 mM) for 24 h under hypoxia (1% O_2_), were collected and prepared for cytometric analysis of cell cycle distribution. B & C. HepG2 cells treated with Q6 (10 μM) in the presence or absence of caffeine (2 mM) for 24 h under hypoxia (1% O_2_). Detection of apoptosis by flow cytometry (B) and caspase cascade by Western blot (C) were then performed. D & E. HepG2 cells treated with Q6 in the presence or absence of ATM specific inhibitor KU-60019 (3 μM) under hypoxia (1% O_2_), and subjected to sub-G1 analyses (D) and Western blot analyses (E), respectively. F. Western blot was used to assess the role of ATM during apoptosis induced by Q6 in hypoxia. HepG2 cells were treated with ATM RNAi or vector RNAi in the presence or absence of Q6 (5 μM) under hypoxia (1% O_2_) condition.

The therapeutic effects of the topo II—targeted drugs were partially attributable to apoptosis. Our preliminary data have showed that Q6 could induce apoptosis of hepatocellular carcinoma via caspase-cascade in both time and dose dependent manners. As members of phosphatidylinositol 3-kinase-related kinase family, ATM plays critical roles during the DNA damage response[22]. In order to explore the role of DNA DSBs and ATM/ATR pathway activation induced by Q6, western blot analysis were used to assess the effect of Q6 on ATM/ATR signaling pathways. It is noteworthy that in HepG2 (Fig 4A) and Bel-7402 (Fig 4B) cancer cells, Q6 (2.5 μM, 5 μM) under hypoxia for 6 h could activate ATM signaling pathway in a dose-dependent manner. In the contrast, the ATR-Chk1 pathway was not activated under the same experimental conditions (Fig 4A and 4B). Thus we introduced caffeine[13] (pre-incubation, 2 mM, 30 min) and KU-60019[23,24] (pre-incubation, 3 μM, 30 min), two ATM kinase inhibitors, to further explore the role of ATM signaling pathway. Caffeine pretreatment could significantly abate the G2-M arrest caused by Q6 in hepatocellular carcinoma, which suggested Q6 induced G2-M arrest was caused by DNA DSBs which were sensed by ATM pathway (Fig 5A). To further explore DNA DSBs and ATM pathway activation in apoptosis provoked by Q6, caffeine and KU-60019 were further utilized to block the ATM kinase, Fig 5B showed that caffeine could aggravate Q6-induced apoptosis. In consistent with these findings, caffeine-pretreatment sensitized HepG2 and Bel-7402 cells to Q6-triggered caspase cascade, as indicated by the extended cleavage of caspase-3 and PARP (Fig 5C). Similarly, KU-60019 preincubation enhanced Q6-caused apoptosis from 25% to 62% (Fig 5D), accompanied with increased activation of caspase cascade (Fig 5E). In order to further verify whether ATM signaling pathway was essential to Q6-induced apoptosis, RNA interference approach was employed. Knockdown of ATM in Q6-treated HepG2 cells led to robust increased protein level of bax (Fig 5F), a pro-apoptotic factor[25]. In conclusion, these data revealed that, as an novel candidate for topo II poisons, Q6 induces apoptosis by accumulating DNA DSBs. Meanwhile, the DNA damage sensor ATM is integrated into this process, and the depletion of ATM will assist cancer cells toward apoptotic destiny.

## Discussion

Our previous study revealed that, Q6, a novel analogue of TPZ, was capable of targeting hypoxic cancer cells, leading to the autophagic degradation of HIF-1α in the hypoxic HCC cells, with improved anti-cancer efficiency than its parental compound TPZ[4]. However, the mechanism(s) of action of Q6 remained to be fully elucidated, since the interruption of HIF-1α by this compound may not sufficiently induce apoptosis, which could not explain the massive cell death when HCC cells were exposed to Q6. The present study unraveled that, in addition to the HIF-1α-targeting effects, the interfering of topo II may also contribute to the anticancer activities possessed by Q6.

Herein, our data displayed that the direct interaction of Q6 with topo II facilitated the inhibitory effects of Q6 on the catalytic activity of topo II, and the stabilization of the topo II-DNA-compound ternary complexes, which resulted in DNA DSBs, cell cycle arrest and ultimately the apoptotic cell death of HCC cells. Despite TPZ and the chemical modulated analogue Q6 shared all these features, the chemical manipulation preferentially endowed Q6 with better topo II–inhibition ability, stronger apoptosis-induction capacity, and improved antitumor efficiency both in vitro and in vivo, according to both the current study and our previous findings[3,4]. Importantly, our results demonstrated that the DSBs triggered by the topo II-poisoning mediated the apoptosis of HCC cells, thus underling the indispensable roles of topo II-targeting in Q6-exerted anticancer effects.

Mounting evidences established the casual link between hypoxia and the negative prognosis of cancer patients, since hypoxia contributed significantly to chemoresistance[26], angiogenesis[27], invasiveness[28,29], resistance to cell death[5,30], which ultimately leading to failed therapy. In order to combat with hypoxia, variety strategies have been developed[2,31]. The most established approach is the induction of HAPs which were designed to specifically eliminate hypoxic tumor cells. As the leading compound of HAPs, TPZ exhibited hypoxic selectivity in a variety of cancer cell models; nevertheless, it has been hampered in randomized phase II and III clinical trials, at least partially, owing to limited improvement in tumor control[32,33]. Thus a number of HAPs other than TPZ have been developed to exploit hypoxia, including PR-104, TH-302 and SN30000, which were undergoing the clinical or preclinical studies[32,34–36]. The substantial efforts to develop novel HAPs are aiming at improve the efficacy to kill hypoxic cancer cells. In this context, the understanding of the mechanisms of action that those HAPs exert under hypoxia may lead to more efficient targeting of the hypoxic tumor environment, which can assist in the rational development of novel hypoxia selective candidates.

The majorities of HAPs described to date are designed to release DNA damaging cytotoxin and thus killed cancer cells[37]. Among these HAPs, PR-104 and NLCQ-1 are DNA cross-linker and intercalator, respectively[38,39]; while AQ4N as well as TPZ were revealed to be topo II poisons[8,40]. In addition to the cytotoxicity-mediated cancer cell killing, the exploitation of dual mode action, namely, simultaneously leading to cell death and interrupting some unique hypoxic cellular target(s), would open the new opportunities to combat with the hypoxia.

Given the critical roles that HIF-1α played under hypoxia with its ability to trans-activate a variety of target genes promoting angiogenesis, metastasis, resistance, proliferation and anti-apoptosis, the suppression of HIF-1α is regarded as a effective way to alleviate the hypoxia-mediated malignancy[6]. Our previous study revealed that Q6 could induce autophagic degradation of HIF-1α, which was mediated by the ubiquitin-binding adaptor protein, SQSTM1/p62[4]. Of note is the factor that, accelerated degradation of HIF-1α could give rise to the inhibition of angiogenesis and metastasis, but may not sufficiently lead to cell death in a short period. In this context, the topo II-targeting effects revealed by our present study raised the notion that Q6 exerted a dual mode of action to exploit HIF-1α and topo II simultaneously, thus achieved a superior anti-cancer activity in hypoxic cancer cells.

Several lines of evidence implicated the interaction of HIF-1α and topo II: Creighton-Gutteridge *et al*. demonstrated that NSC 644221 inhibited hypoxic induction of HIF-1α and the target gene VEGF mRNA expression in U251 cells in a topo II-dependent way, since the silencing of topo IIα, but not topo I, by specific small interfering RNA completely blocked the ability of NSC 644221 to inhibit HIF-1α[41]. In the contrast, another study showed that the topo II-targeting mitoxantrone, but neither doxorubicin nor etoposide (VP-16), could strongly inhibited HIF-1α expression under hypoxic conditions in a dose- and time-dependent manner, through a translation inhibition mechanism. And the mitoxantrone-mediated inhibition of HIF-1α expression was largely independent of two topo II isozymes[42]. Similarly, a novel topo II inhibitor MFTZ-1 reduced HIF-1α accumulation driven by hypoxia or growth factors in human cancer cells, possibly through the inhibition of PI3K-Akt and MAPK pathways, eliciting anti-angiogenesis independently of its topo II inhibition[43]. Thus the suppression of HIF-1α by topo II inhibitors may origins from either topo II-dependent or independent-manners, mainly by blocking the transcriptional or translational levels of HIF-1α. Nevertheless, Q6 is the first agent that could promote the autophagic degradation of HIF-1α in hypoxic cancer cells. And the link between the autophagic degradation of HIF-1α and the topo II suppression warrant further investigation.

ATM is the member of the phoshpoinositide kinases–related protein family, which senses the DNA DSBs and triggers subsequent cell cycle arrest and apoptosis[22]. By arresting cells at specific cell phases, damaged DNA was allowed to be repaired and the cancer cells may survive. Caffeine, an ATM kinase inhibitor, could abolish the DNA damage–induced cell cycle arrest and sensitize the cancer cells to DNA damage[44,45]. In consistent with these findings, the ability to sensitize cancer cells of caffeine was also achieved when it was co-incubated with Q6, as indicated by attenuated cell cycle arrest and enhanced apoptosis. Thus we were encouraged to conclude that the DNA DSBs, origins from the topo II poisoning, initiate the apoptotic cell death in Q6-exposed cancer cells.

In summary, our present study have shown for the first time, that Q6 targets topo II by stabilizing the topo II-DNA-drug ternary complexes, particularly in those hypoxic cells. Thereby Q6 induces DNA DSBs, and leads to apoptotic cell death of the cancer cells under hypoxia. Together with our previous findings, the appreciable hypoxic selective anticancer activity of Q6 could be attributable to its dual mode of action, namely, the topo II-targeting effect as well as the induction of autophagic degradation of HIF-1α. These features favors Q6 a promising HAP drug candidate endowed with improved ability to kill hypoxic cancer cells and deserving of further study and development.

## Supporting Information

S1 Fig Topo II-targeting effect of Q6 in Bel-7402 cells. Bel-7402 cells were exposed to compounds as indicated in the figure, then followed by nuclear extraction, and the subsequent Topo II assay was performed. pHOT-1 DNA was introduced as substrate for Topo II.
(TIF)

S2 Fig Q6 possessed better anticancer activities than TPZ under hypoxia. Trypan blue exclusion staining was used to evaluate apoptosis of HepG2 (A) and Bel-7402 (B) exposed to Q6 (5 μM) or TPZ (5 μM) for 48 hours under normoxia and hypoxia, respectively. Percentages of trypan blue positive (%) in different groups were presented.
(TIF)

S3 Fig Q6 induced G2/M arrest was mediated by ATM in hypoxia. HepG2 (A) and Bel-7402 (B) cells treated with Q6 (5 μM) in the presence or absence of caffeine (2 mM) for 24 h under hypoxia (1% O_2_). Then, the cell were collected and prepared for cytometric analysis of cell cycle distribution. The percentages of the cell population in different phases of cell cycle were analyzed by CELL Quest.
(TIF)

## References

[1] Raleigh SM, Wanogho E, Burke MD, McKeown SR, Patterson LH (1998) Involvement of human cytochromes P450 (CYP) in the reductive metabolism of AQ4N, a hypoxia activated anthraquinone di-N-oxide prodrug. Int J Radiat Oncol Biol Phys 42: 763–767. 9845092 10.1016/s0360-3016(98)00308-3

[2] Wilson WR, Hay MP (2011) Targeting hypoxia in cancer therapy. Nat Rev Cancer 11: 393–410. 10.1038/nrc3064 21606941

[3] Hu Y, Xia Q, Shangguan S, Liu X, Hu Y, Sheng R (2012) Synthesis and biological evaluation of 3-aryl-quinoxaline-2-carbonitrile 1,4-di-N-oxide derivatives as hypoxic selective anti-tumor agents. Molecules 17: 9683–9696. 10.3390/molecules17089683 22890172 PMC6268107

[4] Liu XW, Cai TY, Zhu H, Cao J, Su Y, Hu YZ, et al (2014) Q6, a novel hypoxia-targeted drug, regulates hypoxia-inducible factor signaling via an autophagy-dependent mechanism in hepatocellular carcinoma. Autophagy 10: 111–122. 10.4161/auto.26838 24220190 PMC4389865

[5] Semenza GL (2004) Intratumoral hypoxia, radiation resistance, and HIF-1. Cancer Cell 5: 405–406. 15144945 10.1016/s1535-6108(04)00118-7

[6] Semenza GL (2003) Targeting HIF-1 for cancer therapy. Nat Rev Cancer 3: 721–732. 13130303 10.1038/nrc1187

[7] Smart DJ, Halicka HD, Schmuck G, Traganos F, Darzynkiewicz Z, Williams GM (2008) Assessment of DNA double-strand breaks and gammaH2AX induced by the topoisomerase II poisons etoposide and mitoxantrone. Mutat Res 641: 43–47. 10.1016/j.mrfmmm.2008.03.005 18423498 PMC2581813

[8] Peters KB, Brown JM (2002) Tirapazamine: a hypoxia-activated topoisomerase II poison. Cancer Res 62: 5248–5253. 12234992

[9] Kawatani M, Takayama H, Muroi M, Kimura S, Maekawa T, Osada H (2011) Identification of a small-molecule inhibitor of DNA topoisomerase II by proteomic profiling. Chem Biol 18: 743–751. 10.1016/j.chembiol.2011.03.012 21700210

[10] Sudan S, Rupasinghe HP (2014) Flavonoid-enriched apple fraction AF4 induces cell cycle arrest, DNA topoisomerase II inhibition, and apoptosis in human liver cancer HepG2 cells. Nutr Cancer 66: 1237–1246. 10.1080/01635581.2014.951733 25256427

[11] Zheng L, Yang W, Zhang C, Ding WJ, Zhu H, Lin NM, et al (2011) GDC-0941 sensitizes breast cancer to ABT-737 in vitro and in vivo through promoting the degradation of Mcl-1. Cancer Lett 309: 27–36. 10.1016/j.canlet.2011.05.011 21664043

[12] Zheng L, Fu Y, Zhuang L, Gai R, Ma J, Lou J, et al (2014) Simultaneous NF-kappaB inhibition and E-cadherin upregulation mediate mutually synergistic anticancer activity of celastrol and SAHA in vitro and in vivo. Int J Cancer 135: 1721–1732. 10.1002/ijc.28810 24615207

[13] Zhu H, Huang M, Yang F, Chen Y, Miao ZH, Qian XH, et al (2007) R16, a novel amonafide analogue, induces apoptosis and G2-M arrest via poisoning topoisomerase II. Mol Cancer Ther 6: 484–495. 17308047 10.1158/1535-7163.MCT-06-0584

[14] Tang Y, Chen R, Huang Y, Li G, Huang Y, Chen J, et al (2014) Natural compound Alternol induces oxidative stress-dependent apoptotic cell death preferentially in prostate cancer cells. Mol Cancer Ther 13: 1526–1536. 10.1158/1535-7163.MCT-13-0981 24688053 PMC4165548

[15] Magdolenova Z, Drlickova M, Henjum K, Runden-Pran E, Tulinska J, Bilanicova D, et al (2015) Coating-dependent induction of cytotoxicity and genotoxicity of iron oxide nanoparticles. Nanotoxicology 9 Suppl 1: 44–56. 24228750 10.3109/17435390.2013.847505

[16] Klein F, Laroche T, Cardenas ME, Hofmann JF, Schweizer D, Gasser SM (1992) Localization of RAP1 and topoisomerase II in nuclei and meiotic chromosomes of yeast. J Cell Biol 117: 935–948. 1315786 10.1083/jcb.117.5.935 PMC2289479

[17] Tanabe K, Ikegami Y, Ishida R, Andoh T (1991) Inhibition of topoisomerase II by antitumor agents bis(2,6-dioxopiperazine) derivatives. Cancer Res 51: 4903–4908. 1654204

[18] Liu LF (1989) DNA topoisomerase poisons as antitumor drugs. Annu Rev Biochem 58: 351–375. 2549853 10.1146/annurev.bi.58.070189.002031

[19] Hsieh T, Lee MP, Brown SD (1994) Structure of eukaryotic type I DNA topoisomerase. Adv Pharmacol 29A: 191–200. 7826858 10.1016/s1054-3589(08)60546-3

[20] Chen AY, Liu LF (1994) DNA topoisomerases: essential enzymes and lethal targets. Annu Rev Pharmacol Toxicol 34: 191–218. 8042851 10.1146/annurev.pa.34.040194.001203

[21] Rogakou EP, Pilch DR, Orr AH, Ivanova VS, Bonner WM (1998) DNA double-stranded breaks induce histone H2AX phosphorylation on serine 139. J Biol Chem 273: 5858–5868. 9488723 10.1074/jbc.273.10.5858

[22] Shiloh Y (2001) ATM and ATR: networking cellular responses to DNA damage. Curr Opin Genet Dev 11: 71–77. 11163154 10.1016/s0959-437x(00)00159-3

[23] Zirkin S, Davidovich A, Don J (2013) The PIM-2 kinase is an essential component of the ultraviolet damage response that acts upstream to E2F-1 and ATM. J Biol Chem 288: 21770–21783. 10.1074/jbc.M113.458851 23760264 PMC3724634

[24] Biddlestone-Thorpe L, Sajjad M, Rosenberg E, Beckta JM, Valerie NC, Tokarz M, et al (2013) ATM kinase inhibition preferentially sensitizes p53-mutant glioma to ionizing radiation. Clin Cancer Res 19: 3189–3200. 10.1158/1078-0432.CCR-12-3408 23620409 PMC3687028

[25] Cartron PF, Oliver L, Martin S, Moreau C, LeCabellec MT, Jezequel P, et al (2002) The expression of a new variant of the pro-apoptotic molecule Bax, Baxpsi, is correlated with an increased survival of glioblastoma multiforme patients. Hum Mol Genet 11: 675–687. 11912183 10.1093/hmg/11.6.675

[26] Graeber TG, Osmanian C, Jacks T, Housman DE, Koch CJ, Lowe SW, et al (1996) Hypoxia-mediated selection of cells with diminished apoptotic potential in solid tumours. Nature 379: 88–91. 8538748 10.1038/379088a0

[27] Liao D, Johnson RS (2007) Hypoxia: a key regulator of angiogenesis in cancer. Cancer Metastasis Rev 26: 281–290. 17603752 10.1007/s10555-007-9066-y

[28] Chang Q, Jurisica I, Do T, Hedley DW (2011) Hypoxia predicts aggressive growth and spontaneous metastasis formation from orthotopically grown primary xenografts of human pancreatic cancer. Cancer Res 71: 3110–3120. 10.1158/0008-5472.CAN-10-4049 21343390

[29] Hill RP, Marie-Egyptienne DT, Hedley DW (2009) Cancer stem cells, hypoxia and metastasis. Semin Radiat Oncol 19: 106–111. 10.1016/j.semradonc.2008.12.002 19249648

[30] Gammon L, Mackenzie IC (2015) Roles of hypoxia, stem cells and epithelial-mesenchymal transition in the spread and treatment resistance of head and neck cancer. J Oral Pathol Med. 10.1111/jop.12327 25952002

[31] Melillo G (2007) Targeting hypoxia cell signaling for cancer therapy. Cancer Metastasis Rev 26: 341–352. 17415529 10.1007/s10555-007-9059-x

[32] Hicks KO, Siim BG, Jaiswal JK, Pruijn FB, Fraser AM, Patel R, et al (2010) Pharmacokinetic/pharmacodynamic modeling identifies SN30000 and SN29751 as tirapazamine analogues with improved tissue penetration and hypoxic cell killing in tumors. Clin Cancer Res 16: 4946–4957. 10.1158/1078-0432.CCR-10-1439 20732963 PMC3390971

[33] Marcu L, Olver I (2006) Tirapazamine: from bench to clinical trials. Curr Clin Pharmacol 1: 71–79. 18666379 10.2174/157488406775268192

[34] Abbattista MR, Jamieson SM, Gu Y, Nickel JE, Pullen SM, Patterson AV, et al (2015) Pre-clinical activity of PR-104 as monotherapy and in combination with sorafenib in hepatocellular carcinoma. Cancer Biol Ther 16: 610–622. 10.1080/15384047.2015.1017171 25869917 PMC4622463

[35] Saggar JK, Tannock IF (2014) Activity of the hypoxia-activated pro-drug TH-302 in hypoxic and perivascular regions of solid tumors and its potential to enhance therapeutic effects of chemotherapy. Int J Cancer 134: 2726–2734. 10.1002/ijc.28595 24338277

[36] Sun JD, Liu Q, Ahluwalia D, Li W, Meng F, Wang Y, et al (2015) Efficacy and safety of the hypoxia-activated prodrug TH-302 in combination with gemcitabine and nab-paclitaxel in human tumor xenograft models of pancreatic cancer. Cancer Biol Ther 16: 438–449. 10.1080/15384047.2014.1003005 25679067 PMC4623012

[37] Ahn GO, Brown M (2007) Targeting tumors with hypoxia-activated cytotoxins. Front Biosci 12: 3483–3501. 17485316 10.2741/2329

[38] Patterson AV, Ferry DM, Edmunds SJ, Gu Y, Singleton RS, Patel K, et al (2007) Mechanism of action and preclinical antitumor activity of the novel hypoxia-activated DNA cross-linking agent PR-104. Clin Cancer Res 13: 3922–3932. 17606726 10.1158/1078-0432.CCR-07-0478

[39] Papadopoulou MV, Bloomer WD (2003) NLCQ-1 (NSC 709257): exploiting hypoxia with a weak DNA-intercalating bioreductive drug. Clin Cancer Res 9: 5714–5720. 14654556

[40] Patterson LH, McKeown SR (2000) AQ4N: a new approach to hypoxia-activated cancer chemotherapy. Br J Cancer 83: 1589–1593. 11104551 10.1054/bjoc.2000.1564 PMC2363465

[41] Creighton-Gutteridge M, Cardellina JH 2nd, Stephen AG, Rapisarda A, Uranchimeg B, Hite K, et al (2007) Cell type-specific, topoisomerase II-dependent inhibition of hypoxia-inducible factor-1alpha protein accumulation by NSC 644221. Clin Cancer Res 13: 1010–1018. 17289897 10.1158/1078-0432.CCR-06-2301

[42] Toh YM, Li TK (2011) Mitoxantrone inhibits HIF-1alpha expression in a topoisomerase II-independent pathway. Clin Cancer Res 17: 5026–5037. 10.1158/1078-0432.CCR-11-0235 21653687

[43] Dai M, Miao ZH, Ren X, Tong LJ, Yang N, Li T, et al (2010) MFTZ-1 reduces constitutive and inducible HIF-1alpha accumulation and VEGF secretion independent of its topoisomerase II inhibition. J Cell Mol Med 14: 2281–2291. 10.1111/j.1582-4934.2009.00822.x 19538463 PMC3822569

[44] Wang Q, Fan S, Eastman A, Worland PJ, Sausville EA, O'Connor PM (1996) UCN-01: a potent abrogator of G2 checkpoint function in cancer cells with disrupted p53. J Natl Cancer Inst 88: 956–965. 8667426 10.1093/jnci/88.14.956

[45] Yao SL, Akhtar AJ, McKenna KA, Bedi GC, Sidransky D, Mabry M, et al (1996) Selective radiosensitization of p53-deficient cells by caffeine-mediated activation of p34cdc2 kinase. Nat Med 2: 1140–1143. 8837615 10.1038/nm1096-1140

