# Supplementary figures and images for: Hypoxia-Targeted Drug Q6 Induces G2-M Arrest and Apoptosis via Poisoning Topoisomerase II under Hypoxia
Source: PLoS One. 2015 Dec 9;10(12):e0144506. doi: 10.1371/journal.pone.0144506 (PMC4674137; doi:10.1371/journal.pone.0144506)

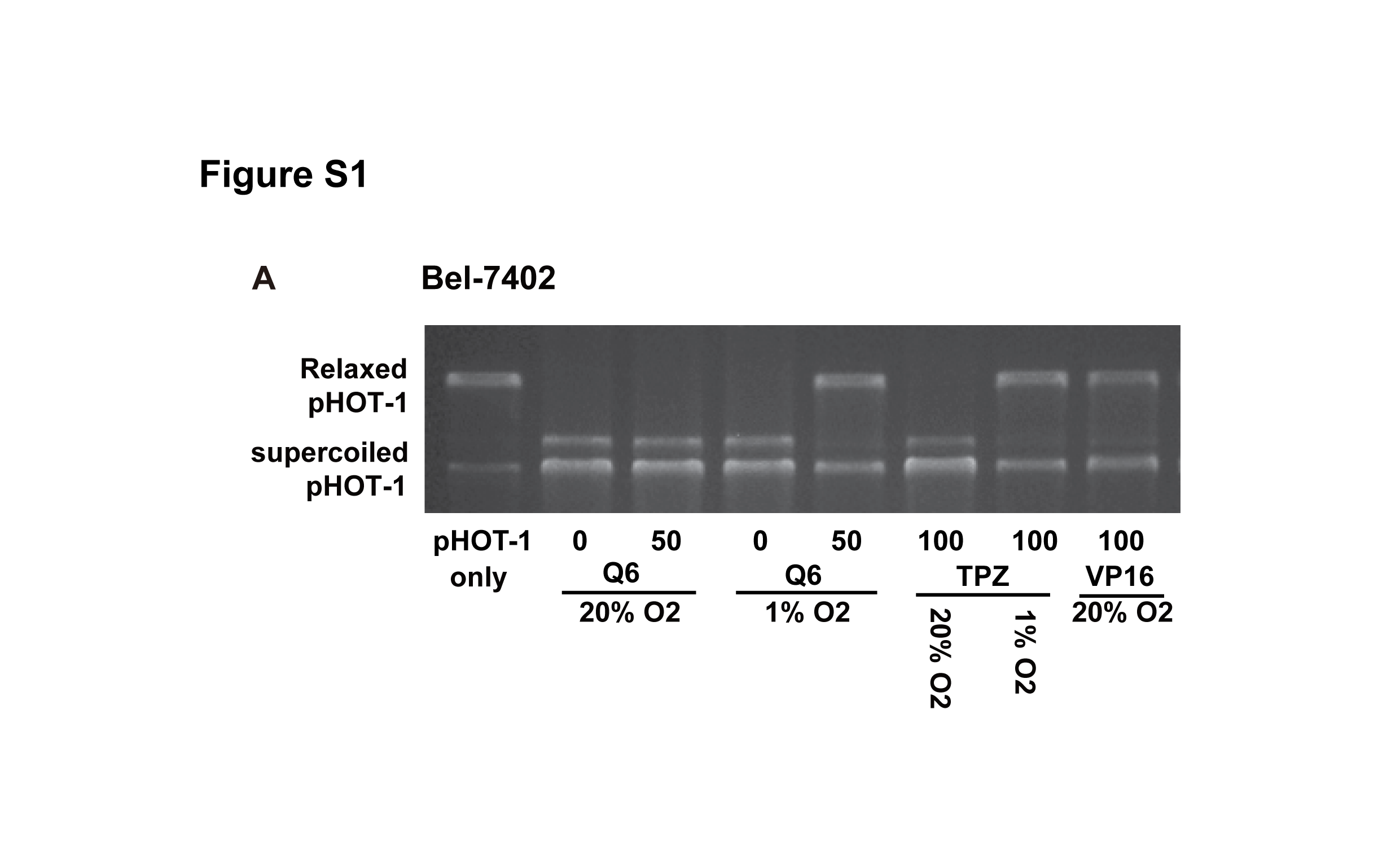

Supplement: S1 Fig — Bel-7402 cells were exposed to compounds as indicated in the figure, then followed by nuclear extraction, and the subsequent Topo II assay was performed. pHOT-1 DNA was introduced as substrate for Topo II. (TIF) [file pone.0144506.s001.tif]

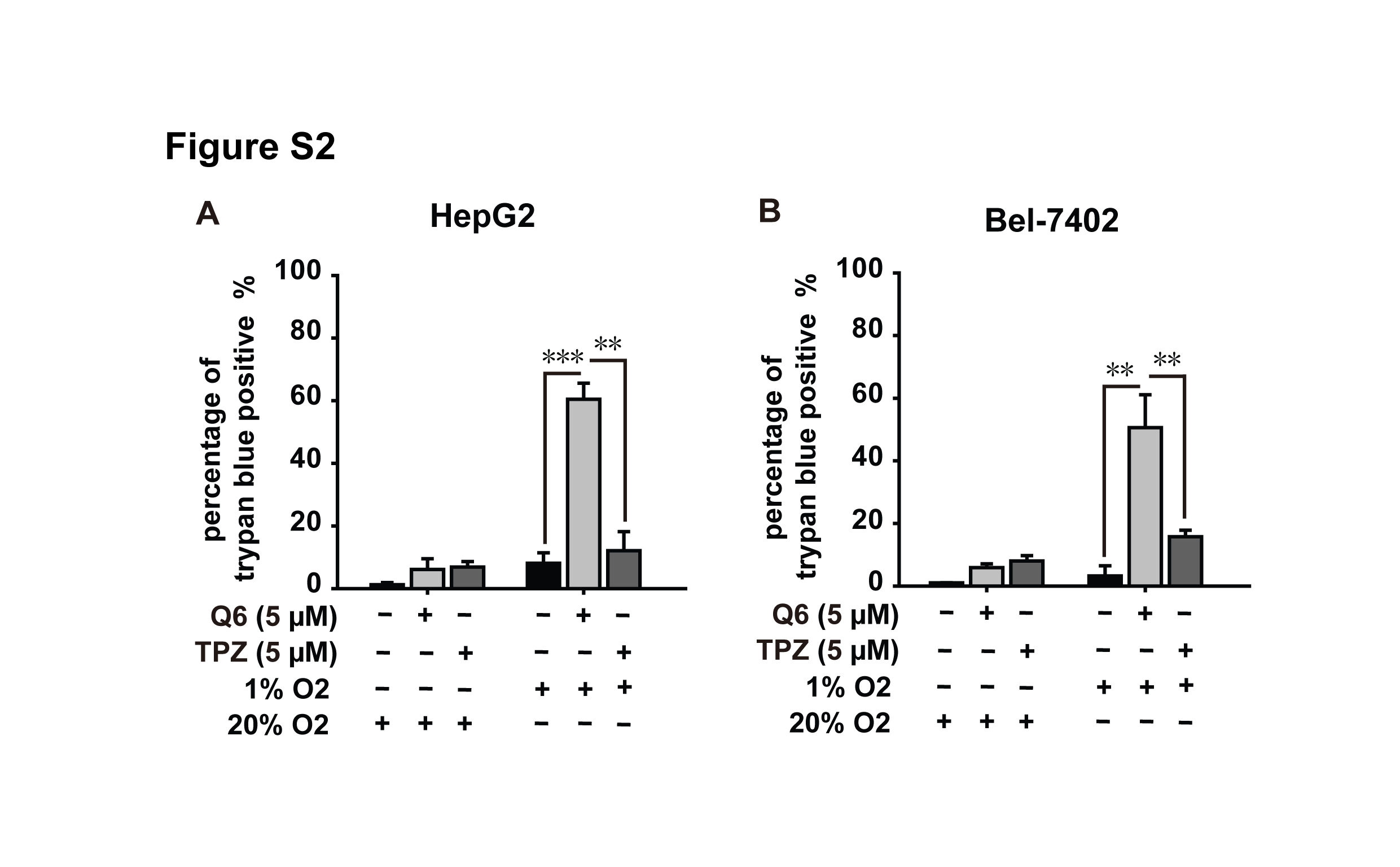

Supplement: S2 Fig — Trypan blue exclusion staining was used to evaluate apoptosis of HepG2 (A) and Bel-7402 (B) exposed to Q6 (5 μM) or TPZ (5 μM) for 48 hours under normoxia and hypoxia, respectively. Percentages of trypan blue positive (%) in different groups were presented. (TIF) [file pone.0144506.s002.tif]

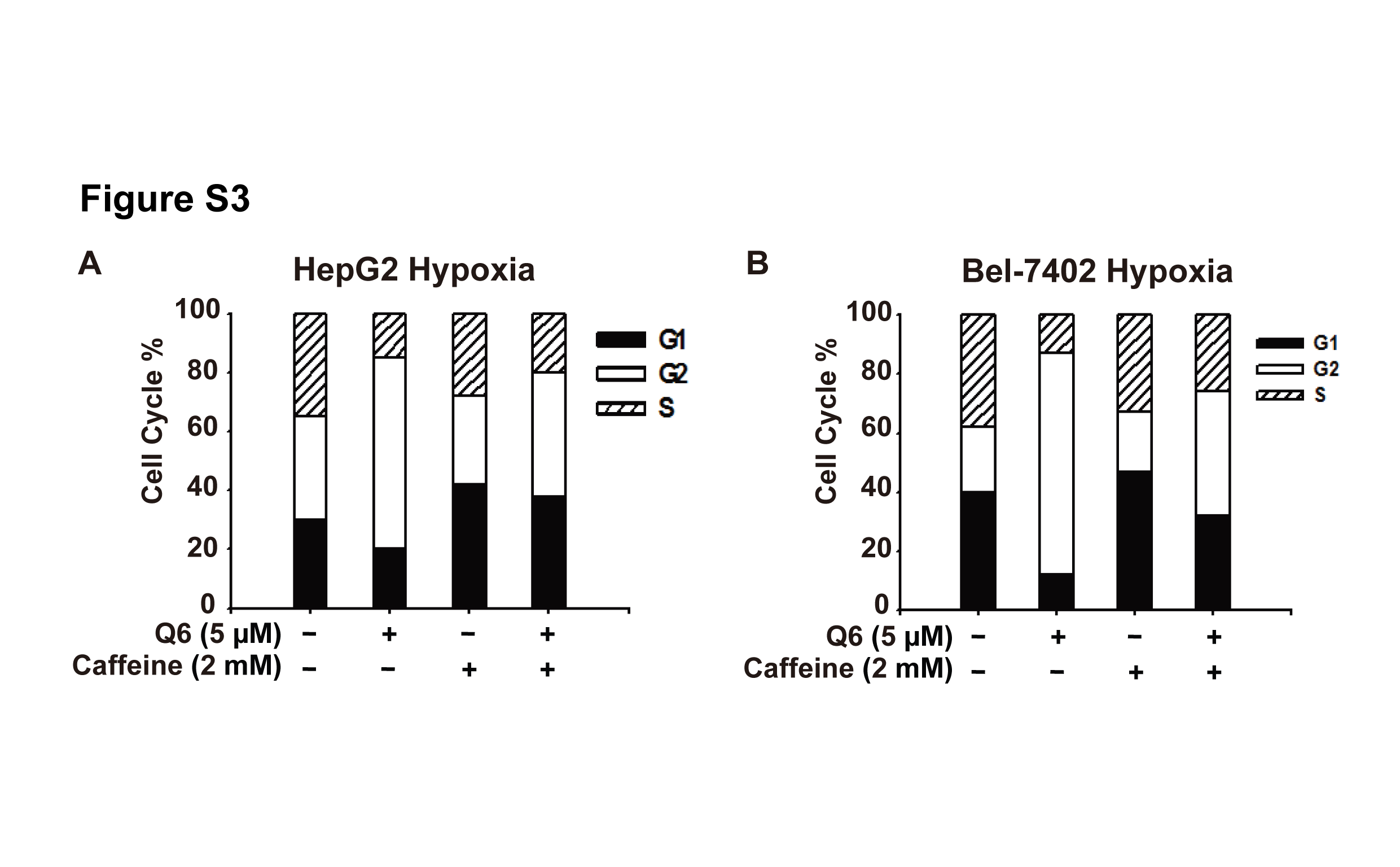

Supplement: S3 Fig — HepG2 (A) and Bel-7402 (B) cells treated with Q6 (5 μM) in the presence or absence of caffeine (2 mM) for 24 h under hypoxia (1% O2). Then, the cell were collected and prepared for cytometric analysis of cell cycle distribution. The percentages of the cell population in different phases of cell cycle were analyzed by CELL Quest. (TIF) [file pone.0144506.s003.tif]
